# Popeye's sign: A diagnostic clue

**DOI:** 10.1002/ccr3.7378

**Published:** 2023-05-20

**Authors:** Takayuki Yamada

**Affiliations:** ^1^ Asunaro Clinic Takasaki Japan

**Keywords:** arm injuries, humerus, shock, tendon injuries

## Abstract

**Key Clinical Message:**

Sudden onset bulging of the upper arm may indicate biceps tendon rupture.

**Abstract:**

We describe a 72‐year‐old man with Popeye's sign. The patient experienced a sudden shock in his right humerus while mowing grass with his right arm using wide sweeps of a scythe. His right upper arm had an obvious bulging appearance after 3 days, indicating a rupture of the biceps tendon.

## Graphical abstract

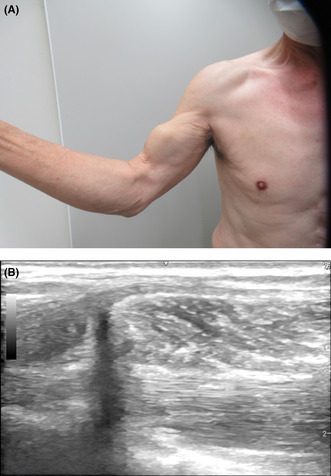

A 72‐year‐old man visited our clinic with a large bulge on his right upper arm. He felt a sudden shock in his right humerus while mowing grass with his right arm using wide sweeps of a scythe. Although there was no pain, his right upper arm had an obvious bulging appearance after 3 days (Figure 1A). Ultrasonography revealed no hematoma or tumor. Additionally, the bulging biceps muscle was shown to separate from the humeral articulation and did not move with arm curl or stretch (Figure 1B). The presented images clearly reveal a rupture of the biceps brachii tendon, called “Popeye's sign.” Since his muscle power showed no bilateral difference, conservative management was undertaken. Sudden onset bulging of the upper arm may indicate biceps tendon rupture.

**FIGURE 1:**
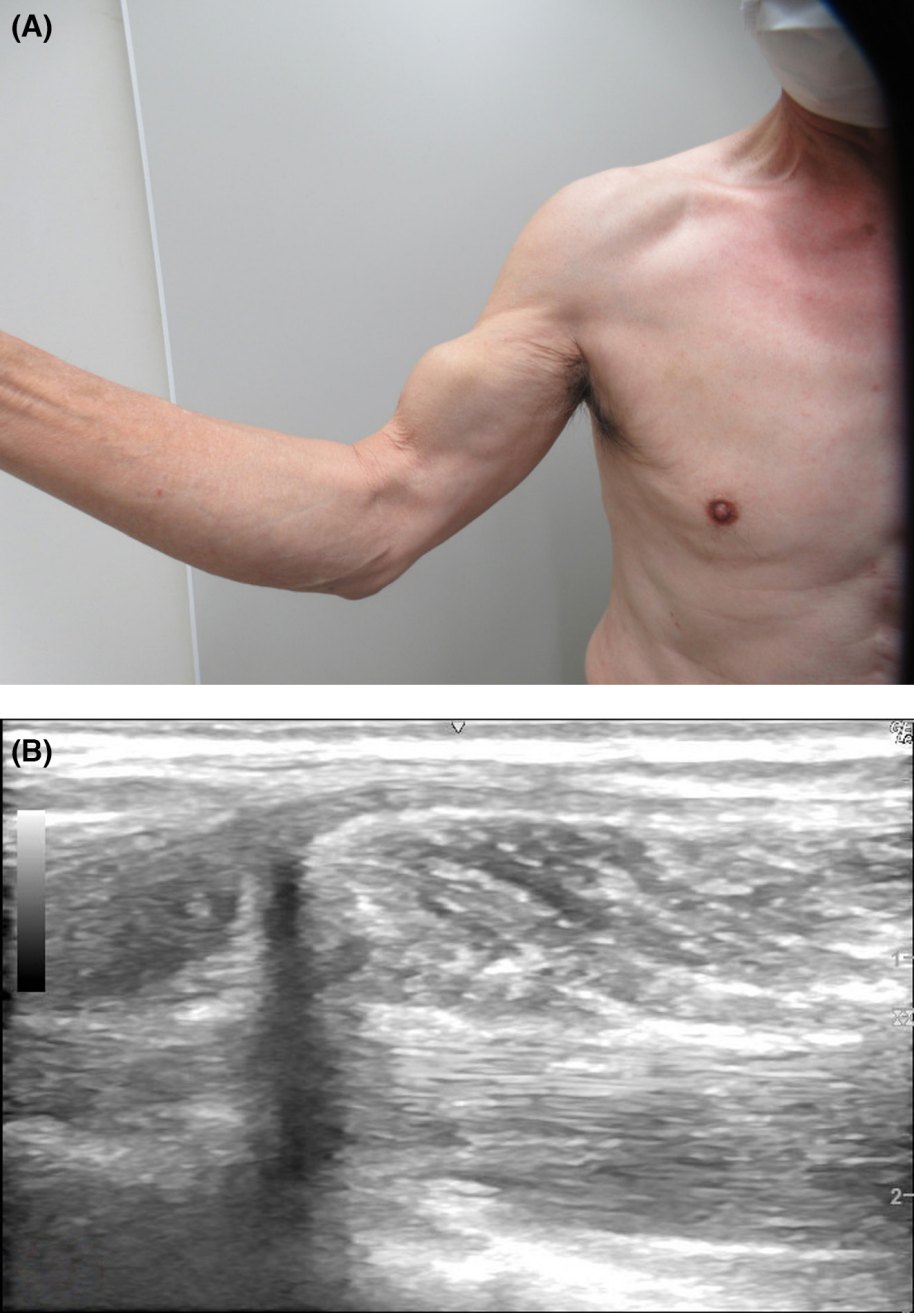
(A): Right upper arm with an obvious bulging appearance. (B): Ultrasonography findings showing no hematoma or tumor. The bulging biceps muscle is shown separating from the humeral articulation and not moving with arm curl or stretch.

Bulging of the biceps muscle rarely occurs clinically after rupture of the biceps tendon. Conservative management is often sufficient for most of these cases, as the tendon rupture does not interfere with normal life.^1^, ^2^

## AUTHOR CONTRIBUTIONS

**Takayuki Yamada:** Resources; visualization; writing – original draft.

## FUNDING INFORMATION

No funding was received for this study.

## CONFLICT OF INTEREST STATEMENT

The author declares no conflicts of interest for this article.

## ETHICS STATEMENT

Ethics approval was not required for this study.

## CONSENT STATEMENT

The patient provided written informed consent for publication.

## Data Availability

Data openly available in a public repository that issues datasets with DOIs.
